# Genetic variants in the serum amyloid A2 (*SAA2*) gene as a potential marker for milk production traits in Chinese Holstein cows

**DOI:** 10.1002/vms3.796

**Published:** 2022-04-26

**Authors:** Sayed Haidar Abbas Raza, Chengcheng Liang, Ahmed Mohajja Alshammari, Bandar H. Aloufi, Linsheng Gui, Rajwali Khan, Linsen Zan

**Affiliations:** ^1^ State Key Laboratory of Animal Genetics Breeding & Reproduction, College of Animal Science and Technology Northwest A&F University Yangling P. R. China; ^2^ National Beef Cattle Improvement Center Northwest A&F University Yangling P. R. China; ^3^ Faculty of Science, Biology Department University of Hail Hail Saudi Arabia; ^4^ State Key Laboratory of Plateau Ecology and Agriculture Qinghai University Xining P. R. China; ^5^ Department of Livestock Management, Breeding and Genetics The University of Agriculture Peshawar Pakistan

**Keywords:** genetic variability, milk production traits, SAA2 gene, single nucleotide polymorphism

## Abstract

**Background:**

This study was conducted to detect potential polymorphisms of the serum amyloid A2 (*SAA2*) gene and explore their relationships with milk production traits in Chinese Holstein cows.

**Objectives: This study used:**

sequencing technology conducted in 532 Chinese Holstein cows.

**Methods:**

Three single nucleotide polymorphisms (SNPs) were identified within intron 1, named g.14061A>G, g.14072G>C and g.14819C>T. Eight estimated haplotypes were identified, of which three major haplotypes had a frequency of Hap3 (‐ACC‐), Hap5 (‐GCC‐) and Hap2 (‐AGT‐), with 17.9%, 12.30% and 8.10%, respectively.

**Results:**

The association analysis of single markers (g.14061A>G and g.14819C>T) and combined genotypes (Hap1/5) revealed prominent effects on milk production traits in Chinese Holstein cows (*p* < 0.05).

**Conclusions:**

Our results suggest that the *SAA2* gene is associated with economic traits in Chinese Holstein cows and may be used as candidate gene for marker‐assisted selection and management in breeding programs.

## Graphical abstract

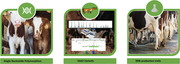

## 1 INTRODUCTION

In mammals, serum amyloid A (SAA) is comprised of five isoforms with high conservation and various biological functions, designated as *SAA1*, *SAA2*, *SAA3*, *M‐SAA3.2* and *SAA4*, respectively (Kovačević‐Filipović et al., 2012). SAA is synthesized primarily by hepatocytes, macrophages, vascular endothelial cells and adipocytes (Ahmed et al., 2012; Artl et al., 2000). Clinical studies confirmed that SAA is considered a biomarker of inflammation and angiocardiopathy, with dramatic increases in SAA plasma levels in response to inflammatory and atherosclerotic cardiovascular diseases (Smole et al., 2020). The *SAA2* gene encodes the amphipathic alpha‐helical apolipoprotein (Chait et al., 2005) that plays a pivotal role in mobilization of cholesterol during tissue repair and regeneration and was described as a ‘gatekeeper’ for its function in normal tissues (Urieli‐Shoval et al., 2000). When released, the *SAA2* gene readily bound to high‐density lipoprotein (HDL), and then rerouted HDL transport (Benditt & Eriksen, 1977). Via modulating the ability of HDL, the *SAA2* gene contributed to reverse transport of cholesterol and removed excess cholesterol from the body (De Beer et al., 2014). Compared to cows with lower milk protein and fat percentage, expression of the *SAA2* gene was remarkably reduced in the mammary gland with extremely high milk protein and fat percentage (Cui et al., 2014). Therefore, these findings lend credence to the hypothesis that the *SAA2* gene is an excellent candidate marker for milk composition traits in dairy cattle.

The purpose of the present research was to analyze the influence of *SAA2* gene polymorphisms on the comparison of milk traits in Chinese Holstein cows and to determine the changes in milk traits during lactation depending on polymorphisms of the analyzed genes.

## 2 MATERIALS AND METHODS

### 2.1 Animals and phenotypic data

A total of 532 Chinese Holstein dairy cows belonging to 24 sire families were collected from Xi'an Caotan Animal Husbandry Co. LTD (Shaanxi Province, China). All of the cows were fed with the same regular total mixed ration composed of concentrated feed and coarse fodder and were routinely milked three times a day (07:00, 15:00, 23:00). The milk yields were recorded with a computerized herd management system. The dairy herd improvement (DHI) in milk samples were provided from Xi'an Caotan Animal Husbandry Co. LTD.

### 2.2 DNA isolation

Genomic DNA from blood samples were collected by TIANamp Blood DNA kit (Tiangen, Beijing, China). The quality of DNA was measured using a NanoDrop ND‐2000c Spectrophotometer (Thermo Scientific).

### 2.3 PCR amplification and single nucleotide polymorphism detection

According to the sequence of the bovine *SAA2* gene (GenBank accession no. AC_000186.1), four pairs of PCR primers were designed with Primer Premier Software (Version 4.0). Detailed information of primers is depicted in Table 1. PCR was performed in a 30 μl reaction mixture containing Mix (TaKaRa, Dalian, China) 15.0 μl, upstream primer 0.6 μl, downstream primer 0.6 μl, ddH_2_O 11.8 μl, DNA 2.0 μl. PCR conditions were as follows: initial denaturation at 95°C for 5 min, followed by 35 cycles at 94°C for 30 s; annealing temperature for 30s; 72°C for 40 s; and a final extension at 72°C for 10 min, and then sequenced using an ABI 3730 sequencer (ABI, Foster City, CA, USA).

**TABLE 1:** Primers used for DNA sequencing for serum amyloid A2 (*SAA2*) gene

Name	Primer Sequence (5′–3′)	Temperature (°C)	Product length	Amplified region
P1	GGTCCTCCAGGAGAATGTGA	58.5	527 bp	Part of intron 1
	GTGCAGGGTCAGATGTGAGA			
P2	TCACATCTGACCCTGCACTC	61.5	503 bp	Part of intron 1
	AGTCTCTCAGCCATGCGTTT			
P3	AAACGCATGGCTGAGAGACT	57.5	516 bp	Part of intron 1
	GACAGGGGTGAGGAGAACAG			

Gene frequencies and Hardy–Weinberg equilibrium (HWE) were computed using POPGENE software (Version 3.0). The extent of linkage disequilibrium (LD) between the identified single nucleotide polymorphisms (SNPs) was estimated using Haploview 3.32 software (Broad Institute of MIT and Harvard, Cambridge, MA, USA) (Raza, Shijun, et al., 2020; Raza, Khan, et al., 2020; Raza, Liu, 2020; Wei et al., 2018).

### 2.4 Statistical analysis

The associations of the SNPs and milk production traits were analyzed by analysis of variance (ANOVA) the SPSS 16.0 software (IBM Company, NY, USA). The equation was as follows: *Y_ijklm_* = *μ* + *Y_i_* + *G_j_* +*L_k_* +*S_l_* + *E_ijklm_*, where *Y_ijklm_* is the trait observation, *μ* is the overall mean, *Y_i_* is the fixed effect of the *i*th year (*i* = 1, 2, 3 and 4), *G_j_* is the fixed effect of the *j*th genotype (*j* = 1, 2 and 3), *L_k_* is the fixed effect of the *k*th calving age (*k* = 1, 2, 3 and 4), *S_l_* is the random effect of the *l*th sire (*l* = 1, 2, 3, 4 and 5), and *E_ijklm_* is the random error.

The additive, dominance, and allele substitution effects of single SNPs were evaluated by SPSS 16.0 software (IBM Company, NY, USA). The equation is as follows:

$$\mathrm{Additive}=\left(\text{homozygous}\;\text{genotypes}\;1-\text{homozygous}\;\text{genotypes}\;2\right)/2;$$

$$\begin{array}{ccc} \mathrm{Dominance} & = & \text{heterozygous}\;\text{genotype}-(\text{homozygous}\;\text{genotypes}\;1\\ & & +\;\text{homozygous}\;\text{genotypes}\;2)/2; \end{array}$$

$$\begin{array}{ccc} & & \text{Allele}\;\text{substitution}=\mathrm{additive}+\mathrm{dominance}\;(\text{allele}\;\text{frequencies}\;\text{of}\\ & & \;\text{homozygous}\;\text{genotypes}\;1-\text{allele}\;\text{frequencies}\;\text{of}\;\text{homozygous}\\ & & \;\text{genotypes}\;2). \end{array}$$

## 3 RESULTS AND DISCUSSION

### 3.1 Polymorphisms and genetic diversity

**FIGURE 1:**
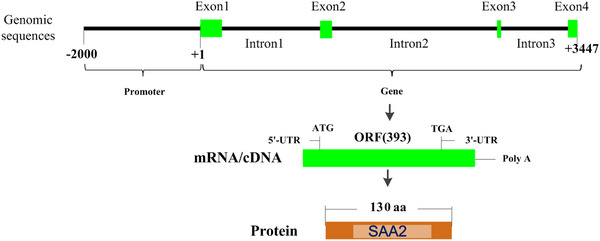
Structure of serum amyloid A2 (*SAA2*) gene. *Source*: https://www.ncbi.nlm.nih.gov/gene/506412

The bovine SAA2 gene is located on chromosome 29 of the bovine genome. The total length of SAA2 is 3447 bp, comprising the genomic coordinates starting from 26458422 to 26461868 (NC_037356.1, Reference genome bos taurus published in GenBank in the NCBI database). This gene comprises 04 exons, the ORF that started from the start codon to the stop codon is 393 bp, and the putative protein contains 131 amino acids (Figure 1).

In the current study, three SNPs, including g.14061A>G, g.14072G>C and g.14819C>T, were identified by DNA direct sequencing. All three loci mapped on intron 1 of the *SAA2* gene. The genotype TT of g.14819C>T was not observed in the sampled population, probable explanations may be that (1) this genotype at g.14819C>T locus may never exist in the population and (2) the experimental population size was insufficient to capture full genetic variation (Gui et al., 2015).

### 3.2 Genetic diversity analysis

Table 2 lists the genotype frequencies, maximum allele frequency, PCI and HWE of *SAA2* gene in Chinese Holstein dairy cows. AA of g.14061A>G was the most prevalent genotype (64.29%). GG of g.14072G>C was more frequent (54.70%) compared to the others genotype. CC was the most prevalent genotype (73.87%) at g.14819C>T locus. The *χ* ^2^ test indicated that the g.14061A>G and g.14072G>C were in HWE (*p* < 0.05), while the g.14819C>T was severely out of HWE (P > 0.05). It was speculated that the g.14819C>T loci carried out significance selection pressure (Lee et al., 2008), resulting in the loss of certain alleles, except those for the favoured traits (Huang et al., 2011).

**TABLE 2:** Population genetic analysis of single nucleotide polymorphisms (SNPs) in serum amyloid A2 (*SAA2*) gene

Loci (gene)	Genotypic frequency (%)			Maximum allele frequency (%)	HWE
g.14061A>G	AA	AG	GG		
	64.29	29.70	6.02	79.14 (A)	*p* > 0.05
g.14072G>C	GG	GC	CC		
	54.70	37.59	7.71	73.50 (G)	*p* > 0.05
g.14819C>T	CC	CT	TT		
	73.87	26.13	–	86.94 (C)	*p* < 0.05

Abbreviation: HWE, Hardy–Weinberg equilibrium.

### 3.3 Haplotypes analysis

As presented in Table 3, eight estimated haplotypes were detected in the Chinese Holstein dairy cows. The frequency of Hap1 haplotype (─AGC─) was 51.00%, followed by Hap3 haplotype (─ACC─), Hap5 haplotype (─GCC─) and Hap2 haplotype (─AGT─), with 17.9%, 12.30% and 8.10%, respectively. High‐frequency haplotypes probably presented at the sampled population long ago, which may be directly or indirectly regulated by different rearing environments (Sawyer et al., 2006). Due to the absence of statistical significance, the Hap4 (─ACT─), Hap6 (─GGT─) and Hap8 (─GCT─) were ignored (frequency < 5.00%).

**TABLE 3:** Frequencies analysis of serum amyloid A2 (*SAA2*) gene haplotypes

Haplotype	g.14061A>G	g.14072G>C	g.14819C>T	Frequency (%)
Hap1	A	G	C	51.00
Hap2	A	G	T	8.10
Hap3	A	C	C	17.90
Hap4	A	C	T	2.10
Hap5	G	G	C	12.30
Hap6	G	G	T	2.00
Hap7	G	C	C	5.60
Hap8	G	C	T	1.00

### 3.4 Association analysis

So far, litter polymorphism on the *SAA2* gene is reported regarding herbivore breeding and reproduction. Previously, two mutations (c.17G>C and c.114G>A) within the 5′‐regulatory region of the *SAA2* gene were found to be statistically significance for milk yield, fat yield and protein yield in Chinese Holstein cows as a result of alteration in *SAA2* gene transcriptional activity (Yang et al., 2015). In addition, both c.‐84 G>C and c.114G>A were detected in the *SAA2* gene of Polish Holstein‐Friesian cattle. Individuals with the CC genotype of c.‐84 G>C were characterized by the highest content of protein and the lowest content of fat in milk (Kowalewska‐Luczak et al., 2021). In the current study, association analysis between SNPs of the *SAA2* gene and milk production traits (Table 4), as well as, the significant additive effects, dominant effects and allele substitution effects were observed (Table 5). Furthermore, individuals carrying GG genotype at g.14061A>G locus had significantly higher milk fat percentage and milk protein percentage than individuals with AA genotype (*p* < 0.05). Compared to animals with genotype TT, the role of CT genotype at g.3208C>T locus caused the highest average for lactose percentage (*p* < 0.05). Different from previous studies, the location of mutations was found on the introns in the current study. The mutation differences may be explained by the diversity of the sample population.

**TABLE 4:** Association of genotypes of single nucleotide polymorphisms (SNPs) in serum amyloid A2 (*SAA2*) gene with comparison of milk traits in Chinese Holstein cows

Loci	Genotypes (N)	Milk fat percentage (%)	Milk protein percentage (%)	Lactose percentage (%)
g.14061A>G	AA (342)	4.17 ± 0.03^b^	3.26 ± 0.02^b^	5.13 ± 0.01
	AG (158)	4.35 ± 0.04^ab^	3.30 ± 0.02^b^	5.12 ± 0.01
	GG (32)	5.19 ± 0.07^a^	3.77 ± 0.05^a^	5.04 ± 0.03
g.14072G>C	GG (291)	4.27 ± 0.04	3.29 ± 0.02	5.13 ± 0.01
	GC (200)	4.33 ± 0.04	3.32 ± 0.03	5.11 ± 0.01
	CC (41)	4.21 ± 0.08	3.32 ± 0.05	5.13 ± 0.02
g.14819C>T	CC (393)	4.24 ± 0.03	3.29 ± 0.02	5.08 ± 0.01^b^
	CT (139)	4.42 ± 0.05	3.32 ± 0.05	5.24 ± 0.02^a^

*Note*: Values with different superscripts letters are significantly different (*p* < 0.05).

**TABLE 5:** Genetic effects of the three single nucleotide polymorphisms (SNPs) in serum amyloid A2 (*SAA2*) gene on the milk production traits

Loci	Genotype	Milk fat	Milk protein	Lactose
g.14061A>G	Additive	−0.510*	−0.255*	0.045
	Dominant	−0.330	−0.215	0.035
	Allele substitution	−0.760*	−0.438*	0.071
g.14072G>C	Additive	0.030	−0.015	0.000
	Dominant	0.090	0.015	−0.020
	Allele substitution	0.088	−0.009	−0.013
g.14819C>T	Additive	2.120	1.645	2.540*
	Dominant	2.300	1.167	2.700
	Allele substitution	3.160	2.168	4.705*

* significant difference (*p* < 0.05).

Haplotypes composed of SNPs provided greater power than single‐marker analysis for associations of inheritable characters because of the ancestral structure captured in the distribution of haplotypes (Akey et al., 2001). Currently, the analysis of combined haplotypes with milk production traits in Chinese Holstein dairy cows is listed in Table 6. The frequencies of haplotype combinations < 5.0% were not considered. Individuals with Hap1/5 (─AGC─GGC─) had greater milk fat percentage values than those individuals with Hap1/1 (─AGC─AGC─) (*p* < 0.05).

**TABLE 6:** Association of dipotypes of serum amyloid A2 (*SAA2*) gene with comparison of milk traits in Chinese Holstein cows

Dipotypes (N)	Milk fat percentage (%)	Milk protein percentage (%)	Lactose percentage (%)
Hap1/1 (146)	4.09 ± 0.05^b^	3.21 ± 0.02	5.12 ± 0.01
Hap1/2 (51)	4.27 ± 0.08^ab^	3.34 ± 0.04	5.19 ± 0.02
Hap1/3 (84)	4.18 ± 0.06^ab^	3.23 ± 0.03	5.10 ± 0.02
Hap1/5 (54)	4.38 ± 0.07^a^	3.22 ± 0.04	5.10 ± 0.02
Hap1/7 (56)	4.27 ± 0.07^ab^	3.30 ± 0.04	5.09 ± 0.02

*Note*: Values with different superscripts letters are significantly different (*p* < 0.05).

Correlation analysis revealed that both g.14061A>G and g.14819C>T were identified in the intron of the *SAA2* gene and contributed to the genetic breeding of Chinese Holstein cows. Although the structure of the encoded protein was not changed, intronic SNPs may influence assembly of spliceosome components, thereby maintaining the genetic stability (Wu et al., 2019). In addition, intronic SNPs affected the initiation and extension of transcription via binding to several enhancers or cis‐acting elements (Chai et al., 2015). In two Chinese indigenous beef cattle, the g.2694 C>T and g.3801 T>C mapping on intronic SNPs of max dimerization protein 3 (*MXD3*) influenced growth traits (Hao et al., 2020).

The intronic g.18341 C>T of the lipoprotein lipase (*LPL*) gene was significantly correlated with withers height and chest depth in Nanyang cattle (Gui et al., 2016). Currently, it was speculated that 14061A>G and g.3208C>T may impact the biological function, resulting in an alteration in phenotype of mammal.

Milk performance is considered to be primary economic trait in dairy cattle, which is regulated by a series of factors, such as inheritance, age, diet, location and other random environment factors. Accumulating genome‐wide association studies (GWAS) indicated that a numbers of candidate genes (i.e., Toll‐like receptor 4, UDP‐glucose dehydrogenase, complement component 4) are involved in complex mechanisms, thereby altering milk performance traits (Gui et al., 2016; Li et al., 2015; Palombo et al., 2018), Here, we detected significant SNPs within the *SAA2* gene that potentially influence milk composition and yield in Chinese dairy cattle, which could be used as a genetic marker aiming towards long‐term improvement in production.

## 4 CONCLUSION

In summary, three SNPs (g.14061A>G, g.14072G>C and g.14819C>T) and eight haplotypes were discovered within the *SAA2* gene of Chinese Holstein cows. The association analysis of single markers (g.14061A>G and g.14819C>T) and combined genotypes (Hap1/5) revealed prominent effects on comparison of milk traits in Chinese Holstein cows. Further research should be conducted in a large population before they could be applied as genetic markers for selection purposes.

## CONFLICT OF INTEREST

The authors declare no conflict of interest.

## AUTHOR CONTRIBUTION

*Conceptualization, data curation, formal analysis, investigation, software, validation, visualization, writing—original draft and writing—review and editing*: Sayed Haidar Abbas Raza. Conceptualization, data curation, formal analysis, investigation: Chengcheng Liang. *Formal analysis, validation and writing—review and editing*: Ahmed Mohajja Alshammari. *Conceptualization, formal analysis, methodology and writing—review and editing*: Bandar H. Aloufi. *Formal analysis and Writing—review and editing*: Linsheng Gui. *Software, validation and writing —review and editing*: Rajwali Khan. *Funding acquisition, investigation, methodology, project administration, resources, supervision and writing—review and editing*: Linsen Zan.

## ETHICS STATEMENT

The experimental animals were dealt as per standard operating procedures (SOPs) formulated by Chinese Council of Animals Care, and further ap‐proved by ‘Experimental Animal Management Committee Approval Number is (EAMC 2020‐1298)’ of the Northwest Agricultural and Forestry University. The sampling was performed after human killing of the experimental animals at College of Animal Science, Shaanxi, Yangling, China.

### PEER REVIEW

The peer review history for this article is available at https://publons.com/publon/10.1002/vms3.796.

## Data Availability

The data that support the findings of this study are available from the corresponding author upon reasonable request.
